# Extracellular matrix turnover in severe COVID-19 is reduced by corticosteroids

**DOI:** 10.1186/s12931-025-03098-9

**Published:** 2025-01-22

**Authors:** Janesh Pillay, Antine W. Flikweert, Matijs van Meurs, Marco J. Grootenboers, Simone van der Sar-van der Brugge, Peter H. J. van der Voort, Morten A. Karsdal, Jannie M. B. Sand, Diana J. Leeming, Janette K. Burgess, Jill Moser

**Affiliations:** 1 Department of Critical Care, University of Groningen, University Medical Center Groningen, Groningen, The Netherlands; 2 Department of Pathology & Medical Biology, University of Groningen, University Medical Center Groningen, Groningen, The Netherlands; 3 Research Institute for Asthma and COPD, University Medical Center Groningen, University of Groningen, Groningen, The Netherlands; 4 Department of Pulmonary Medicine, Amphia Hospital, Breda, The Netherlands; 5 Nordic Bioscience, Hepatic and Pulmonary Research, Herlev, Denmark

**Keywords:** COVID-19, ARDS, Extracellular matrix, Neo-epitopes, Corticosteroids

## Abstract

**Background:**

Severe and critical COVID-19 is characterized by pulmonary viral infection with SARS-CoV-2 resulting in local and systemic inflammation. Dexamethasone (DEX) has been shown to improve outcomes in critically ill patients; however, its effect on tissue remodeling, particularly collagen turnover, remains unclear. This study investigated the association between circulating extracellular matrix (ECM) remodeling neo-epitopes and COVID-19 severity, their relationship with mortality, and the effect of DEX on these markers.

**Methods:**

We conducted a multi-center prospective cohort study involving 226 COVID-19 patients: 157 with severe disease admitted to the ward and 69 with critical disease admitted to the ICU. Plasma samples were collected at ICU admission and at discharge or death. Circulating collagen degradation (C3M, C4Ma3, and C6M) and synthesis (PRO-C3, PRO-C4, and PRO-C6) neo-epitopes were measured. Longitudinal analysis of ECM neo-epitope changes during ICU stay and their association with mortality was performed, along with an evaluation of the impact of DEX treatment on these markers.

**Results:**

Critically ill patients exhibited higher levels of collagen degradation (reflecting inflammatory driven ECM destruction) (C3M, C6M) and collagen synthesis (strongly related to fibroblast activity) (PRO-C3, PRO-C6) neo-epitopes than severe patients. Increased collagen turnover, measured during ICU stay, was associated with mortality. Non-survivors displayed rising levels of collagen degradation and synthesis markers over time, whereas survivors had stable or declining levels. In non-survivors without DEX treatment, C6M and PRO-C6 levels were significantly increased, whereas these elevations were less pronounced in patients treated with DEX.

**Conclusion:**

Our findings suggest that elevated collagen turnover is associated with poor outcomes in critically ill COVID-19 patients. DEX treatment appeared to attenuate ECM remodeling, although this effect was not linked to improved survival. Further studies are needed to confirm these observations and better understand the role of ECM remodeling in COVID-19 and the potential therapeutic impact of corticosteroids.

**Supplementary Information:**

The online version contains supplementary material available at 10.1186/s12931-025-03098-9.

## Introduction

Severe and critical COVID-19 is characterized by pulmonary viral infection with SARS-CoV-2 resulting in local and systemic inflammation [1]. Extensive research has focused on elucidating the inflammatory pathways involved in COVID-19 [2]. Modulation of inflammation by corticosteroids, such as dexamethasone (DEX), improves the outcome of patients, although the exact mechanism of action remains unclear [3]. The role of tissue remodeling in the pathogenesis of severe COVID-19 and the impact of DEX have not been extensively investigated.

In acute respiratory distress syndrome (ARDS), tissue damage and remodeling occur early following diagnosis [4]. Biomarkers of collagen production, such as NT-Pro-collagen 3, have been associated with poor outcomes in all-cause ARDS [4, 5]. Similarly, in COVID-associated ARDS, this product of collagen synthesis has also been associated with poor outcomes [6]. However, there are many other collagens potentially involved in tissue remodeling in ARDS which are yet to be investigated. In an exploratory study, we demonstrated that circulating extracellular matrix (ECM) fragments are associated with mortality in acute inflammation and sepsis [7]. However, whether tissue remodeling, as reflected by circulating ECM fragments released during disease activity, is responsive to therapeutic interventions remains unclear [8].

The lung is composed of multiple ECM proteins such as collagens, each with distinct functions and locations within the tissue [9]. Tissue damage is accompanied by disruption of the ECM, followed by a remodeling process that includes synthesis, degradation, and post-translational modification of ECM components. Properly balanced remodeling restores tissue integrity, whereas perturbations in this process may lead to either tissue simplification or excess tissue formation such as fibrosis, both of which result in impaired lung function.

During ECM remodeling, fragments from ECM proteins can be released into the circulation [10]. These fragments, identified through detection of neoepitiopes, are generated when proteases, such as matrix-metalloproteinases (MMPs) and a disintegrin and metalloproteinase with thrombospondin motifs (ADAMTS), cleave (pro)-peptides from ECM fibers. These fibers may be either newly synthesized or already integrated into the tissue. As a result, circulating ECM fragments have the potential to serve as easily measurable, non-invasive biomarkers for assessing disease status and activity. It is recognized that measurement of some ECM fragments provides an indication of fibroblast activity in diseased tissues [11, 12]. In several chronic lung diseases, circulating fragments from different collagens have been shown to reflect disease severity, predict progression and response to therapeutic interventions [13, 14].

In critically ill patients, these circulating ECM signatures can provide valuable insights into the balance between tissue degradation and synthesis, potentially indicating ongoing tissue destruction or aberrant healing processes such as excessive fibroproliferation or fibrosis. To explore this, we assessed tissue remodeling in hospitalized patients with differing severities of COVID-19, by measuring several neo-epitopes of ECM fragments associated with collagen degradation and synthesis and assessed their response to dexamethasone therapy.

## Methods

### Study design

**Fig. 1:**
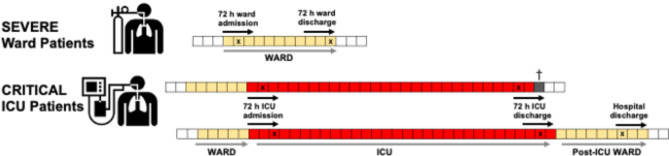
Study design. Plasma was collected from severe COVID-19 patients as early as possible within 48 h of ward admission and again within 72 h prior to hospital discharge. For critical COVID-19 patients, plasma was collected as early as possible within 72 h of ICU admission and again as late as possible within 72 h prior to ICU discharge (or death). When feasible, an additional sample was collected near hospital discharge. The clinical course is represented by squares, with each square denoting a day. Yellow squares indicate the ward stay, red squares indicate the ICU stay, grey box indicates death, and “X” marks the timing of plasma sampling and measurement during hospitalization

In this study, we obtained plasma samples and clinical data from COVID-19 patients admitted to two hospitals in The Netherlands: the University Medical Center Groningen (UMCG) and Amphia Hospital in Breda, as described previously [15, 16]. The inclusion periods were as follows: March 6, 2020, to April 3, 2020, at Amphia Hospital (1st wave); April 24, 2020, to June 6, 2020, at UMCG (1st wave); and September 28, 2020, to December 3, 2020, at UMCG (2nd wave). Patients admitted to the general ward were classified as having ‘severe COVID-19,’ while those admitted to the ICU were classified as having ‘critical COVID-19.’ Plasma samples were collected within 48 h of admission for patients with severe COVID-19 and within 72 h of ICU admission for critical COVID-19 patients (Fig. 1). Additional plasma samples were collected during ICU stay, including before ICU discharge, for patients who were transferred to the general ward or those who died in the ICU. Additionally, plasma was collected from patients shortly before they left the general ward and were discharged from the hospital.

### Severe and critical COVID-19 patients

SARS-CoV-2 infection of patients included in this study was confirmed by RT-PCR analysis of oropharyngeal and nasopharyngeal swabs. Patients received treatment based on local COVID-19 protocols. Routine ICU care involved high-dose anticoagulation with low-molecular-weight heparin (LMWH) (87 IE/kg twice daily) and selective digestive tract decontamination. During the first wave, chloroquine was part of the standard treatment for ward and ICU patients until the Netherlands National Institute for Public Health and Environment recommended the discontinuation of its use by the end of March 2020. Starting in July 2020, all patients admitted during the second wave who required supplemental oxygen therapy were administered dexamethasone 6 mg daily, and some patients also received remdesivir. None of the hospitalized COVID-19 patients had received the SARS-CoV-2 vaccine. Acute kidney injury (AKI) was defined according to the Kidney Disease: Improving Global Outcomes (KDIGO) criteria [17], which consider changes in serum creatinine levels and urine output. Patients were defined as having liver dysfunction if they had one of the following: clinical jaundice, Hyperbilirubinaemia (blood bilirubin level twice the upper limit of the normal range), or an increase in alanine transaminase (ALT) or aspartate transaminase (AST) that is twice the upper limit of the normal range. The requirement for informed consent was waived as the analyses were performed using residual plasma samples collected for clinical purposes. This study was approved by the local medical ethical committee of the University Medical Center Groningen under METc 2020/492, and the Medical Research Ethics Committees United under W20.248, and the Central Research Committee Amphia Hospital under N2020-0380). Clinical trial number: not applicable.

### Data Collection

Clinical and demographic data were extracted from the electronic medical records of hospitalized patients. This information included age, sex, body mass index (BMI), medical history, and the clinical course of COVID-19 during hospital admission. A modified version of the World Health Organization (WHO) electronic Case Report Form (eCRF) was utilized to record data within the Clinical Database Infrastructure Research Electronic Data Capture (REDCap) system.

### Plasma ECM neo-epitope analysis

Residual heparinized plasma was collected from all hospitalized patients immediately after routine analysis and stored at -80 °C until biomarker analysis. Patients with severe and critical COVID-19 included in our cohort were admitted to either the general ward or the ICU approximately 8–10 days after the onset of initial COVID-19 symptoms. Neo-epitopes were quantified using specific competitive enzyme-linked immunosorbent assays (ELISA) and competitive immunoassays on an automated platform (IDS i10), utilizing neo-epitope-specific monoclonal antibodies developed by Nordic Bioscience (Herlev, Denmark). These assays were designed to detect specific MMP-mediated degradation fragments of type III collagen (nordicC3M), type IV collagen alpha 3 chain (nordicC4Ma3), and type VI collagen alpha 1 chain (nordicC6M). Additionally, assays measured ADAMTS-2 mediated release of the N-terminal pro-peptide of type III collagen (nordicPRO-C3), an internal 7 S domain of type IV collagen (nordicPRO-C4), and the C-terminal type VIa3 collagen (nordicPRO-C6, endotrophin). The assays were performed using nordicC3M™, nordicC4Ma3™, nordicC6M™, nordicPRO-C3™, nordicPRO-C4™, and nordicPRO-C6™ kits (Nordic Bioscience) as previously described [18–22]. Concentrations of the ECM fragments were within the range of detection. The rate of change in circulating ECM fragment levels over ICU duration was calculated by determining the difference in protein concentration at ICU admission and ICU discharge and dividing by the ICU length of stay in days. For each ECM fragment (F), the rate of change ΔF=(F_[ICU discharge]_-F_[ICU admission]_)/days in ICU.

### Multi-analyte plasma analysis

Residual heparinized plasma samples were collected from all hospitalized patients immediately after routine clinical analysis and stored at -80 °C until biomarker assessment. Patients with COVID-19 included in the cohort were admitted to either the general ward (severe COVID-19) or ICU (critical COVID-19) approximately 8–10 days after symptom onset. Biomarker profiling was conducted using custom-made Human Luminex xMAP multiplex assays (R&D Systems, Abingdon, UK) following the manufacturer’s protocols. Measurements were performed on a Luminex 200 instrument (Luminex, Austin, TX, USA), and data analysis was completed using xPONENT v4.2 software (Luminex). The assessed biomarkers included markers of inflammation, endothelial dysfunction, coagulation, and organ injury. Concentration values below the lower detection limit were assigned the minimum quantifiable value, while values exceeding the upper detection limit were capped at the maximum quantifiable concentration. IL-2 values were mostly below the detection threshold, and CXCL10 values exceeded the upper limit; therefore, these proteins were excluded from further analysis. Previous findings suggested higher visfatin levels in critical COVID-19 patients compared to severe cases [15], but since many samples were below the detection threshold, visfatin was excluded from the current analysis. To monitor inter-assay variability, three of the seven calibration curve points prepared on the first day of measurement were aliquoted and stored at -80 °C. These aliquots were thawed and used as internal controls during each analysis day, and the analyte coefficient of variation (CV) was calculated as described previously [16].

### Statistical analysis

Continuous variables are expressed as median and interquartile range (IQR) values. The significance of differences between groups were assessed using the Mann-Whitney U test. The Kruskal-Wallis test was used to assess differences across multiple groups. Post-hoc comparisons were conducted using Dunn’s test to account for multiple comparisons. Categorical and dichotomous variables are presented as frequencies and percentages, and differences were evaluated using the chi-squared test and Fisher’s exact test. Spearman’s correlation test was employed to examine correlations between variables. Analyses were performed using SPSS Statistics version 28 (IBM Corp, Armonk, NY, USA) and GraphPad Prism version 10 (Graphpad, Boston, MA, USA). Statistical significance was set at *P* < 0.05.

## Results

### Patient Population

A total of 226 patients were included in the study, comprising 157 patients admitted to the general ward for severe COVID-19 and 69 patients admitted to the ICU for critical COVID-19. Patient demographics are summarized in Table 1. The median patient age was 67 years [57–74], and 58% of the patients were male. The length of hospital stay was longer in patients with critical COVID-19 than in those with severe COVID-19 (17 [11-24] vs. 5 [6-12] days, *p* < 0.001). The incidence of pulmonary embolism, stroke, bacteremia, acute kidney injury, liver dysfunction, and mortality was higher in the critically ill group, reflecting the severity of their condition.

**Table 1:** Demographic and clinical characteristics

Variable	Total COVID-19 patients *n* = 226	Severe COVID-19 Ward *n* = 157	Critical COVID-19 ICU *n* = 69	*p*-value Ward vs. ICU
**Demographics**				
Age (years)	67 [57–74]	67 [57–76]	68 [58–73]	0.575
Male, *n* (%)	131 (58.0)	85 (54.1)	46 (66.7)	0.079
BMI (kg/m^2^)	27.2 [24.6–30.7]	26.8 [24.1–30.8]	27.8 [25.2–30.8]	0.200
**Comorbidity**				
Pulmonary disease, *n* (%)	53 (23.5)	35 (22.3)	18 (26.1)	0.535
Chronic cardiac disease, *n* (%)	61 (27.0)	40 (25.5)	21 (30.4)	0.439
Chronic kidney disease, *n* (%)	22 (9.7)	18 (11.5)	4 (5.8)	0.186
Hypertension, *n* (%)	78 (34.5)	54 (34.4)	24 (34.8)	0.955
Diabetes, *n* (%)	46 (20.4)	35 (22.3)	11 (15.9)	0.275
**Clinical course**				
O2 hospital admission (L/min)	4 [2–6]	3 [2–4]	10 [4–15]	< 0.001
Symptom days before hospitalization	9 [6–12]	9 [6–12]	9 [7–14]	0.408
Hospital length of stay, days	7 [4–14]	5 [6–12]	17 [11–24]	< 0.001
**Treatment**				
Antiviral	43 (19.0)	9 (13.0)	34 (21.7)	0.129
Antibiotics	166 (73.5)	97 (61.8)	69 (100)	< 0.001
Corticosteroids	83 (36.7)	53 (33.8)	30 (43.5)	0.163
Antifungal therapy	17 (7.5)	0 (0)	17 (24.6)	< 0.001
Chloroquine	111 (49.1)	73 (46.5)	38 (55.1)	0.235
**ICU characteristics**				
APACHE II score	n/a	n/a	15 [13–20]	n/a
ICU length of stay, days	n/a	n/a	10 [7–22]	n/a
Duration mechanical ventilation, days	n/a	n/a	9 [6–22]	n/a
PaO2/FiO2 after intubation	n/a	n/a	130 [99–178]	n/a
Prone ventilation	n/a	n/a	46 (66.7)	n/a
Tracheostomy	n/a	n/a	6 (8.7)	n/a
ECLS	n/a	n/a	2 (2.9)	n/a
**Complications**				
Pulmonary Embolism	22 (9.7)	5 (3.2)	17 (24.6)	< 0.001
Pneumothorax	3 (1.3)	1 (0.6)	2 (2.9)	0.222
Stroke	8 (3.5)	1 (0.6)	7 (10.1)	0.001
Cardiac ischemia	9 (4.0)	5 (3.2)	4 (5.8)	0.461
Bacteremia*	9 (4.0)	0 (0)	9 (13.0)	< 0.001
Acute kidney injury**	46 (20.4)	11 (7.0)	35 (50.7)	< 0.001
Renal replacement therapy	13 (5.8)	0 (0)	13 (18.8)	< 0.001
Liver dysfunction***	57 (25.2)	23 (14.6)	34 (49.3)	< 0.001
**Death**	31 (13.7)	0 (0)	31 (44.9)	< 0.001

Data are presented as median [IQR], or *n* and percentage; *p*-values are calculated using Mann Whitney U, Chi-squared test or Fishers Exact Test. APACHE Acute Physiology And Chronic Health Evaluation, BMI body mass index, ECLS Extra Corporeal Life Support, ICU Intensive Care Unit, *positive blood cultures, **increase in serum creatinine by 26.5 umol/L within 48 h, or > 1,5 times baseline within the prior 7 days, ***an increase in blood bilirubin, alanine transaminase or aspartate transaminase that is twice the upper limit of the normal range, n/a- Not applicable

### Collagen turnover is associated with COVID-19 severity and ICU mortality

**Fig. 2:**
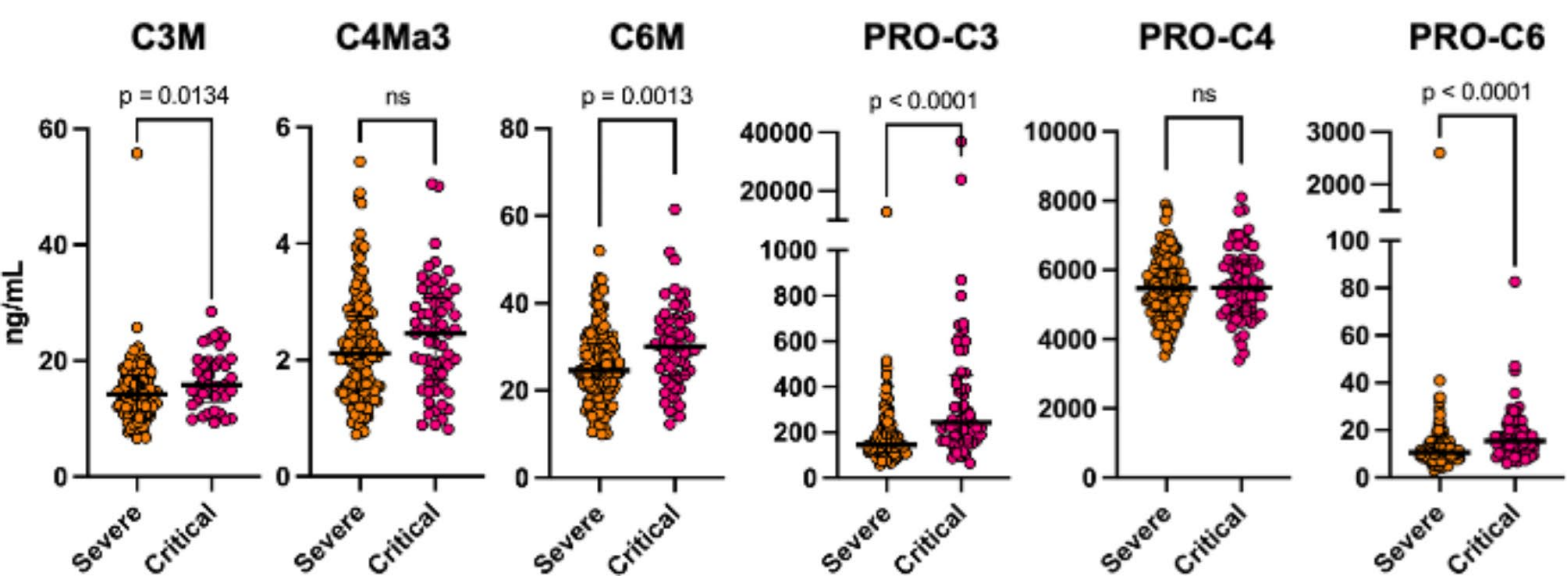
The plasma levels of C3M, C6M, PRO-C3, and PRO-C6 are associated with COVID-19 severity. Circulating neo-epitope levels in critically ill COVID-19 patients (critical COVID-19) and those admitted to the general ward (severe COVID-19) (*n* = 157). Each dot represents an individual patient, and the data are presented as medians with interquartile ranges. Statistical significance was calculated using Mann-Whitney U tests

**Fig. 3:**
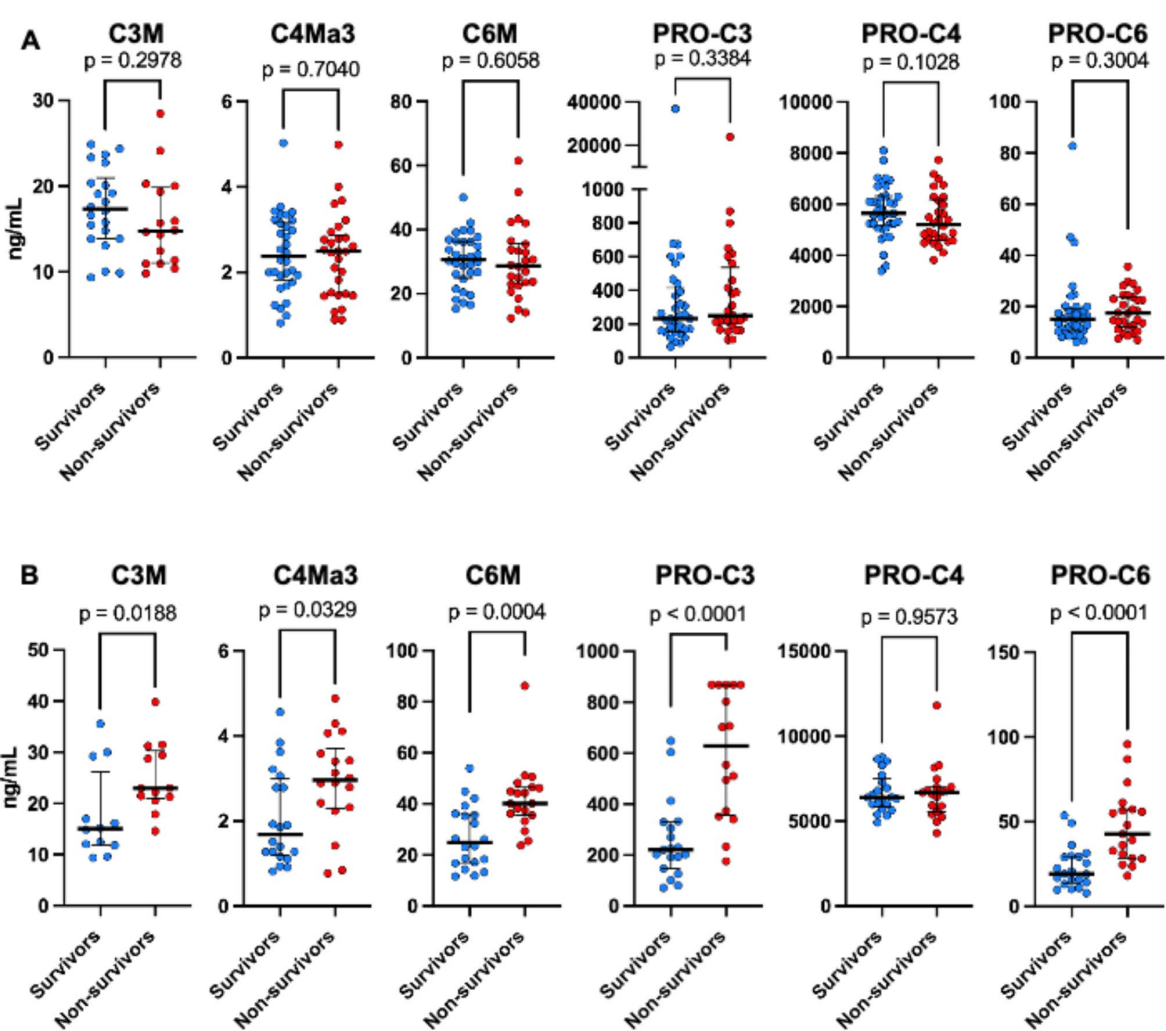
Circulating levels of ECM neo-epitopes at ICU discharge are associated with mortality. **(A)** Circulating levels of ECM neo-epitopes at ICU admission and **(B)** Circulating levels of ECM neo-epitopes at ICU discharge. Each dot represents an individual patient, and the data are presented as medians with interquartile ranges. Statistical significance was calculated using Mann-Whitney U tests

Upon hospital admission, circulating markers of collagen degradation, indicated by the neo-epitopes C3M and C6M, were elevated in patients with critical COVID-19 compared to those with severe COVID-19 (14.3 [11.2–17.6] vs. 15.9 [12.9–20.1] ng/ml, *p* = 0.013) and (24.6 [19.2–30.8] vs. 30.0 [23.4–36.3] ng/ml, *p* = 0.013), respectively (Fig. 2). Collagen synthesis and markers of fibroblast activity, represented by PRO-C3 and PRO-C6, followed a similar pattern, showing higher levels in patients with critical COVID-19 (145.4 [112.9-207.8] vs. 242.3 [168.3–449.0] ng/ml, *p* < 0.0001) and (10.5 [8.1–14.4] vs. 14.5 [11.0-22.5] ng/ml, *p* < 0.0001), respectively. To investigate whether collagen turnover was associated with mortality in critical COVID-19 patients, we analyzed admission samples for collagen neo-epitopes. The mortality rate among critical COVID-19 patients was 45% (Table 1). Those who died were older (71 [63–75] vs. 62 [56–70], *p* = 0.005) but did not differ in terms of underlying chronic disease. Patients who died were more likely to experience serious complications during their ICU stay, including acute kidney injury, stroke, and cardiac ischemia (Table 2). We found that collagen neo-epitopes related to disease activity measured at admission did not significantly differ between survivors and non-survivors (Fig. 3A). However, samples taken near the time of ICU discharge or death in the ICU revealed a higher collagen turnover in non-survivors, except for PRO-C4 (Fig. 3B). These data suggest that higher collagen turnover and dynamic changes during ICU stay are associated with severe disease and poor outcomes.

**Table 2:** Demographic and clinical characteristics: survivors versus non-survivors

Variable	Total ICU patients *n* = 69	Survivors (A) *n* = 38	Non-survivors (B) *n* = 31	*P*-value A versus B
**Demographics**				
Age (years)	68 [58–73]	62 [56–70]	71 [63–75]	0.005
Male, *n* (%)	46 (66.7)	25 (65.8)	21 (67.7)	0.864
BMI (kg/m^2^)	27.8 [25.2–30.8]	27.9 [25.1–31.0]	27.3 [25.2–30.8]	0.791
**Comorbidity**				
Pulmonary disease, *n* (%)	18 (26.1)	10 (26.3)	8 (25.8)	0.962
Chronic cardiac disease, *n* (%)	21 (30.4)	10 (26.3)	11 (35.5)	0.410
Chronic kidney disease, *n* (%)	4 (5.8)	1 (2.6)	3 (9.7)	0.319
Hypertension, *n* (%)	24 (34.8)	12 (31.6)	12 (38.7)	0.536
Diabetes, *n* (%)	11 (15.9)	5 (13.2)	6 (19.4)	0.525
**Clinical course**				
O2 need at hospital admission (L/min)	10 [4–15]	6 [3–15]	14 [5–15]	0.337
Symptom days before hospitalization	9 [7–14]	9 [7–11]	10 [7–14]	0.707
Hospital length of stay, days	17 [11–24]	18 [13–35]	16 [10–24]	0.285
**Treatment**				
Antiviral	34 (21.7)	6 (15.8)	3 (9.7)	0.500
Antibiotics	69 (100)	38 (100)	31 (100)	
Corticosteroids	30 (43.5)	18 (47.4)	12 (38.7)	0.470
Antifungal therapy	17 (24.6)	4 (10.5)	13 (41.9)	0.003
Chloroquine	38 (55.1)	18 (47.4)	20 (64.5)	0.154
**ICU characteristics**				
APACHE II score	15 [13–20]	15 [10–20]	16 [ 15–23]	0.089
ICU length of stay, days	10 [7–22]	10 [7–26]	13 [7–22]	0.999
Duration mechanical ventilation, days	9 [6–22]	8 [5–23]	13 [7–22]	0.389
PaO2/FiO2 after intubation	130 [99–178]	131 [96–199]	127 [99–154]	0.865
Prone ventilation	46 (66.7)	21 (58.3)	25 (80.6)	0.049
Tracheostomy	6 (8.7)	5 (13.9)	1 (3.2)	0.205
ECLS	2 (2.9)	0 (0)	2 (6.5)	0.210
**Complications**				
Pulmonary Embolism	17 (24.6)	7 (18.4)	10 (32.3)	0.185
Pneumothorax	2 (2.9)	1 (2.6)	1 (3.2)	0.999
Stroke	7 (10.1)	1 (2.6)	6 (19.4)	0.040
Cardiac ischemia	4 (5.8)	0 (0)	4 (12.9)	0.036
Bacteremia*	9 (13.0)	2 (5.3)	7 (22.6)	0.068
Acute kidney injury**	35 (50.7)	11 (28.9)	24 (77.4)	< 0.001
Renal replacement therapy	13 (18.8)	5 (13.2)	8 (25.8)	0.181
Liver dysfunction***	34 (49.3)	17 (44.7)	17 (54.8)	0.404

Data are presented as median [IQR], or *n* and percentage; *p*-values are calculated using Mann Whitney U, Chi-squared test or Fishers Exact Test. APACHE Acute Physiology And Chronic Health Evaluation, BMI body mass index, ECLS Extra Corporeal Life Support, ICU Intensive Care Unit, *positive blood cultures, **increase in serum creatinine by 26.5 umol/L within 48 h, or > 1,5 times baseline within the prior 7 days, ***an increase in blood bilirubin, alanine transaminase or aspartate transaminase that is twice the upper limit of the normal range

### Increases in collagen turnover during ICU stay are associated with mortality

**Fig. 4:**
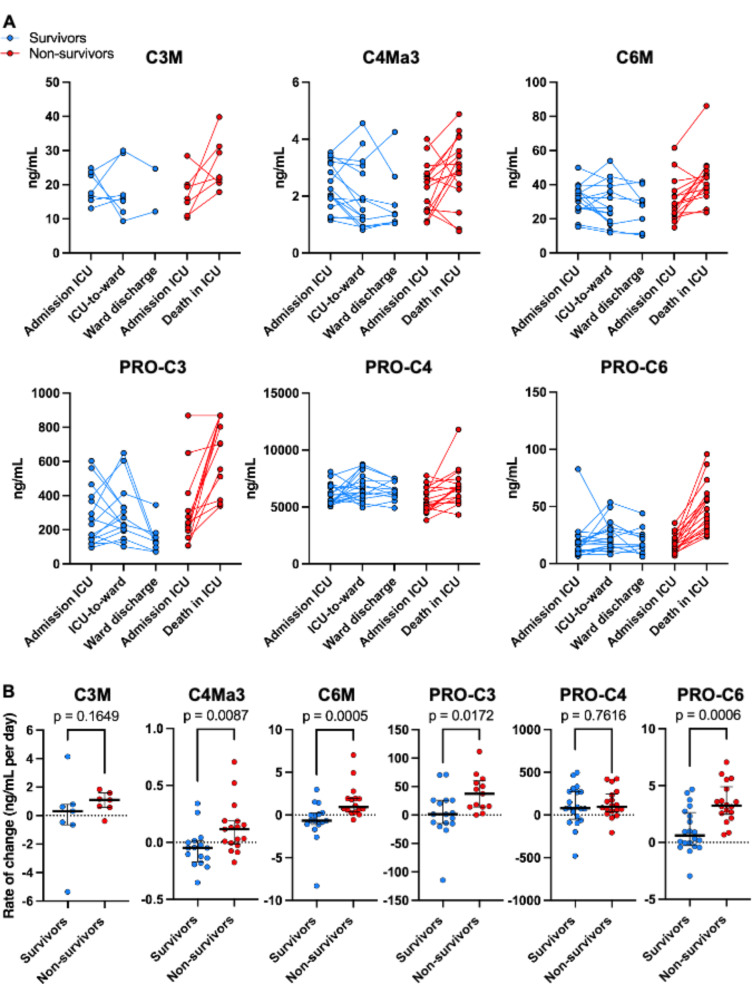
Increased collagen turnover during ICU stay is associated with mortality **(A)** Changes in circulating ECM neo-epitope levels throughout ICU stay and upon discharge to the ward (survivors) or death (non-survivors). **(B)** The rate of change in ECM fragment levels per day. Each dot represents an individual patient, and the data are presented as medians with interquartile ranges. Statistical significance was calculated using Mann-Whitney U tests

We performed a detailed analysis to explore whether alterations in collagen turnover were associated with mortality. While no significant associations were observed in the admission samples, subsequent samples collected during hospitalization demonstrated an association with mortality. The median sampling time in days between ICU admission and discharge was longer in patients who died in the ICU compared to survivors (5 [3-8.8] vs. 9 [5.8–14.0] days, *p* = 0.03). Longitudinal analysis revealed that patients discharged from the ICU to the general ward had stable or decreased collagen turnover, whereas non-survivors (ICU mortality) showed an increase in collagen turnover over time. This pattern was most pronounced for type III and type VI collagen turnover assessed via C3M, C6M, PRO-C3, and PRO-C6 (Fig. 4A). By calculating the daily rate of change between admission and discharge for each collagen neo-epitope, we quantified these differences (Fig. 4B). Daily increases in C4Ma3, C6M, PRO-C3, and PRO-C6 during ICU stay were associated with mortality, whereas stable or decreasing levels of inflammation driven degradation (C3M, C4Ma3, C6M) and fibroblast activity markers (PRO-C3 and PRO-C6) were associated with survival.

### Circulating ECM fragments are not associated with organ injury parameters but are associated with host response biomarkers

**Fig. 5:**
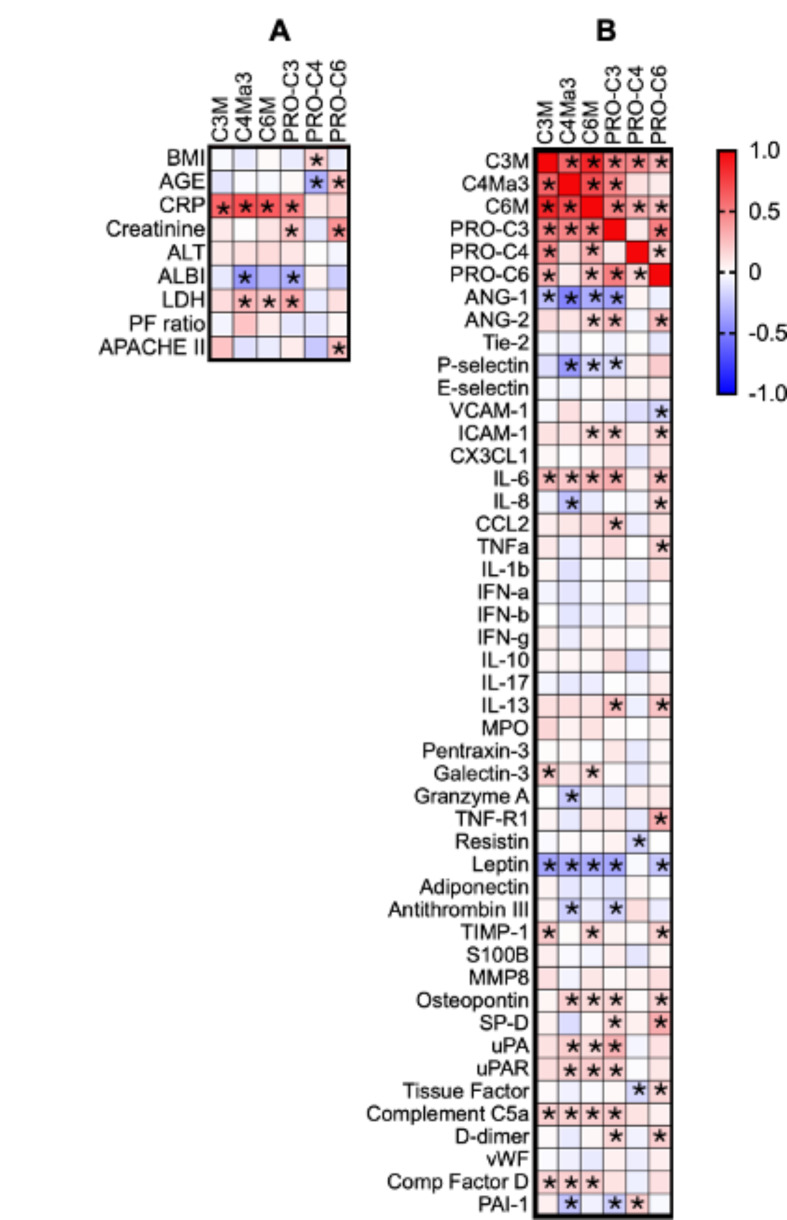
Circulating ECM neo-epitopes are not associated with organ injury parameters but are associated with host response biomarkers. **(A)** Spearman correlation of ECM neo-epitopes, patient demographics, and clinical variables of organ function, injury, and severity of critical illness; **(B)** Spearman correlation of ECM neo-epitopes with markers for inflammation, endothelial dysfunction, and organ injury. * indicates a significant correlation

To further understand the role of ECM fragments in critical COVID-19 progression, we investigated their association with various clinical parameters and host response biomarkers. This analysis aimed to elucidate the potential links between ECM turnover, pulmonary function, critical illness severity, and inflammatory and organ injury biomarkers. ECM neo-epitope levels did not correlate with the severity of pulmonary impairment as measured by the P/F ratio (Fig. 5A). However, surfactant protein-D, a marker of alveolar injury, was associated with PRO-C3 and PRO-C6 levels (Fig. 5B). Although ECM neo-epitopes were linked to mortality and were higher in ICU patients than in patients on the general ward, the APACHE II scores, a measure of critical illness severity, at ICU admission were not associated with ECM neo-epitope levels (Fig. 5A). A strong inverse correlation was observed between Angiopoietin-1 (ANG-1) levels and ECM neo-epitopes, whereas other markers of endothelial dysfunction (Angiopoietin-2, Tie-2, VCAM-1, ICAM-1) showed weak to no correlation (Fig. 5B). Weak to no correlations were found with inflammatory cytokines, with IL-6 showing the strongest (although still weak) correlation. CRP displayed the strongest correlation with all neo-epitopes except for PRO-C4 and PRO-C6 (Fig. 5B).

### Circulating ECM fragments are altered by corticosteroid therapy

**Fig. 6:**
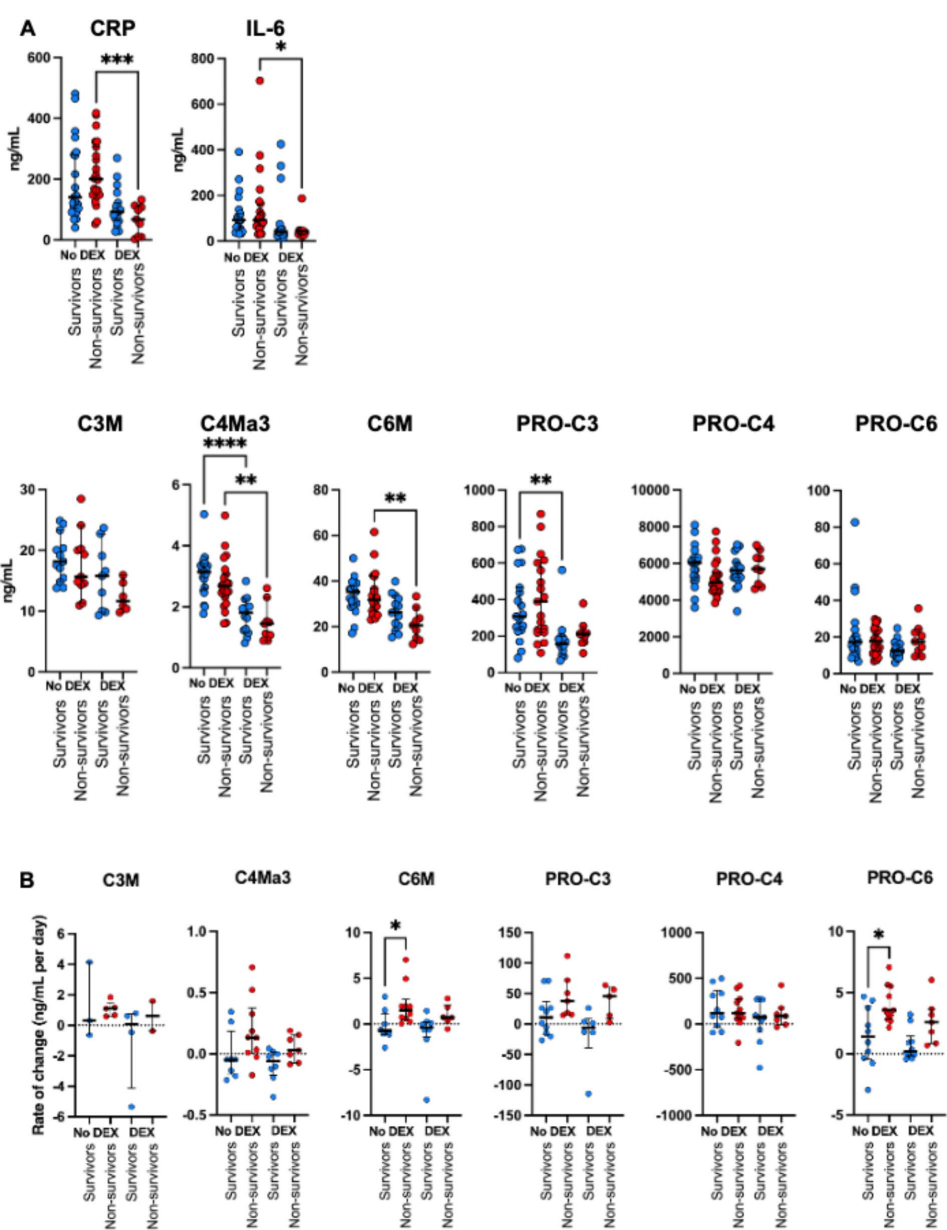
Impact of dexamethasone (DEX) treatment on circulating ECM neo-epitope levels. **(A)** Circulating ECM neo-epitope levels at ICU admission stratified into survivors and non-survivors, and patients who did and did not receive dexamethasone treatment. DEX was given to patients prior to sampling. **(B)** The rate of change in ECM fragment levels per day. Each dot represents an individual patient, and the data are presented as medians with interquartile ranges. The Kruskal-Wallis test was used to assess differences across multiple groups. Post-hoc comparisons were conducted using Dunn’s test to account for multiple comparisons

During patient inclusion, corticosteroid therapy was widely implemented based on the findings of the RECOVERY clinical trial [23]. In our cohort, 25 of 48 ICU patients (52%) received dexamethasone (DEX) prior to the first sampling in the ICU. The mean time between DEX initiation and ICU admission was 1.6 days. As expected, both IL-6 and CRP levels were reduced in patients who received dexamethasone compared with those who did not (41.3 [27.6–49.6] vs. 103.1 [62.6-165.1] ng/ml, *p* = 0.0001) and (82 [49.0-117.5] vs. 198 [109.0-280.0] ng/ml, *p* < 0.0001), respectively (Supplemental Fig. 1A). Interestingly, we found that the circulating levels of C4Ma3 (1.5 [1.1–2.1] vs. 2.9 [2.2–3.4] ng/ml, *p* < 0.0001), C6M (23.1[17.7–30.4] vs. 32.4 [28.4–36.4] ng/ml, *p* = 0.0003), and PRO-C3 (168.9 [136.9-213.2] vs. 311.3 [220.8-601.2] ng/ml, *p* = 0.001) were also reduced in patients that were treated with DEX compared to standard treatment (Supplemental Fig. 1B). When comparing survivors and non-survivors, we found that DEX led to a reduction in circulating neo-epitopes C4Ma3 and PRO-C4 in both survivors and non-survivors and C6M in non-survivors (Fig. 6A). When examining ECM neo-epitope dynamics during the ICU stay, we observed that the daily rate of change varied between patients who received DEX and those who did not (Fig. 6B). In non-survivors without DEX treatment, C6M and PRO-C6 levels showed significant increases, whereas these elevations were less pronounced in those treated with DEX. These findings suggest that DEX treatment attenuates circulating C6M and PRO-C6 levels but that this reduction was not associated with survival outcomes. However, because this exploratory analysis was limited by the small sample size, these results should be confirmed in larger studies to validate the observed effects.

## Discussion

In this study, we showed that circulating ECM fragments strongly related to fibroblast activation and inflammation driven ECM destruction were associated with the severity of COVID-19 and ICU mortality, and that neo-epitopes exposed during the release of C3M, C6M, PRO-C4, and PRO-C6 are reduced following dexamethasone treatment, whereas circulating inflammatory cytokines are not. Consistent with the detection of circulating ECM fragments in COVID-19, we have previously identified ECM fragment neo-epitopes in a human endotoxemia model and in patients with septic shock. In septic shock, elevated circulating ECM neo-epitopes are associated with mortality [7]. In this study, the levels of ECM neo-epitopes were higher in critically ill COVID-19 patients, but their levels at ICU admission were not associated with mortality. ECM fragment neo-epitope levels closer to ICU discharge or death in the ICU (< 72 h) were associated with mortality, as calculated by the increase/decrease per day. This emphasizes the potential value of longitudinal biomarker measurements, in contrast to the commonly employed single-point measurements at ICU admission. Increases in collagen degradation fragments might indicate ongoing tissue damage caused by unresolved or new pulmonary pathologies. Surprisingly, increasing synthesis fragments, possibly involved in tissue healing, were also associated with mortality. This may be due to underlying processes that regulate both synthesis and degradation or increased synthesis leading to excessive fibroproliferation and further deterioration. Further studies on all-cause ARDS are needed to clarify the specific ECM synthesis and degradation profiles and to explore their association with pulmonary and other patient-centered outcomes.

The relationship between inflammation and ECM remodeling remains unclear; however, in this study, only weak correlations between inflammatory cytokines and ECM fragments were observed. Previously, we noted stronger correlations between markers representing these two processes in patients with septic shock, although these correlations remained weak to moderate [7].

Dexamethasone plays a vital role in the treatment of critical COVID-19, reducing mortality by approximately 10% [23], presumably through its anti-inflammatory properties. Corticosteroids have been shown to reduce NT-procollagen III levels in persistent and non-resolving ARDS [24, 25]. Our data indicate that in the acute phase of COVID-19, DEX does not reduce the levels of circulating inflammatory mediators but lowers the levels of several collagen fragments in the circulation. In critically ill patients, inflammation is compartmentalized, meaning that different organs exhibit different inflammatory responses that are not always reflected by circulating mediators. As ECM fragments likely originate from tissues, these biomarkers might provide a better reflection of the compartmentalized tissue response. Although our sampling did not include bronchoalveolar lavage (BAL) fluid, future ECM fragment neo-epitope studies in ARDS should incorporate this, as it would provide a more comprehensive view of lung-specific ECM remodeling. A recent study measured a NT-Procollagen III fragment in the BAL of COVID-19 ARDS patients but did not include other ECM matrix fragments or assess their response to steroids, despite NT-Procollagen III being associated with mortality [6].

Although the exact mechanisms by which DEX improves COVID-19 patient outcomes remain unknown, our findings show that DEX treatment reduces collagen remodeling but does not affect all circulating host inflammatory mediators. The reduction in collagen fragment release by DEX was similar in both survivors and non-survivors. However, this was based on samples taken at ICU admission, where neo-epitope levels did not correlate with mortality. Additionally, the time between DEX administration and the first measurement was relatively short (1.6 days), potentially leading to an underestimation of its effects.

We performed exploratory analyses of daily changes in ECM fragments. The last sampling before ICU discharge or death revealed that increases in collagen synthesis and degradation were blunted in survivors, whereas synthesis and degradation remained ongoing in non-survivors. In addition, DEX appeared to reduce ECM remodeling to levels closer to zero in survivors compared to non-survivors. While these findings are intriguing, they should be interpreted with caution because of the small sample size.

A strength of this study is the inclusion of a large cohort of patients from two different medical centers, enhancing the generalizability of our findings. Moreover, our study design allowed us to monitor patients throughout their ICU stay, enabling us to assess the dynamic changes in ECM fragments detectable in the circulation from ICU admission to later in the general ward and up until discharge from the hospital. However, several limitations should be considered. Although we aimed to collect blood samples at the time of admission, this was not always feasible because of patient transfer from other hospitals and ICUs across the Netherlands. Consequently, for this study, we included samples collected within 48 h of admission to the general ward and within 72 h after admission to the ICU. However, the time between disease onset (positive test) and hospitalization was similar between patients admitted to the ward and ICU [15]. Additionally, we also aimed to collect samples from patients who were as close to ICU and hospital discharge or mortality as possible. However, this was not always possible on the discharge day itself, and as alternative samples were taken as close to discharge as possible, within 72 h. This delay in plasma collection may have influenced the results. Although the study included a sizable patient cohort from two hospitals, ICU mortality was high, and patients were often transferred to other hospitals while still in the ICU. As such, more detailed analyses looking at patient-specific dynamic changes in ECM fragment neo-epitopes in survivors and non-survivors could only be performed in a subgroup of patients and therefore may be limited by the analytical power for conducting a conclusive analysis. Given the exploratory nature of our study and the limited sample size, we employed univariate statistical analyses to identify potential associations while reducing the risk of overfitting. We propose that future research use multivariate statistical methods on larger datasets to further explore these associations and strengthen the clinical interpretation of ECM remodeling, using circulating ECM fragments in acute respiratory distress syndrome. While respiratory system mechanics such as driving pressure, compliance, plateau pressure, and ventilatory settings including PEEP, tidal volume, and respiratory rate were not systematically recorded in this study, we acknowledge their potential relevance to understanding ECM remodeling in critically ill patients. Future studies in patients, but also in mechanical ventilation animal models, should integrate detailed respiratory mechanics and ventilatory settings to provide a more comprehensive perspective on ECM changes in critically ill patients.

## Conclusions

Here, we report that various circulating neo-epitopes reflecting ECM turnover during severe COVID-19 associate with the severity of illness and mortality. In addition, we showed that the release of ECM neo-epitopes are responsive to corticosteroid therapy. Therefore, we conclude that the ECM fragments assessed, reflecting fibroblast activity and inflammation driven tissue destruction, may function as biomarkers for disease severity and steroid responsiveness in patients with ARDS, and that insight into tissue remodeling by longitudinal assessment of ECM fragments might reveal tools for stratification and therapy monitoring in ARDS.

## Electronic supplementary material

Below is the link to the electronic supplementary material.

Supplementary Material 1

## Data Availability

The datasets analysed during the current study are available from the corresponding authors on reasonable request.
