# Supplementary figures and images for: Extracellular matrix turnover in severe COVID-19 is reduced by corticosteroids
Source: Respir Res. 2025 Jan 22;26:32. doi: 10.1186/s12931-025-03098-9 (PMC11755962; doi:10.1186/s12931-025-03098-9)

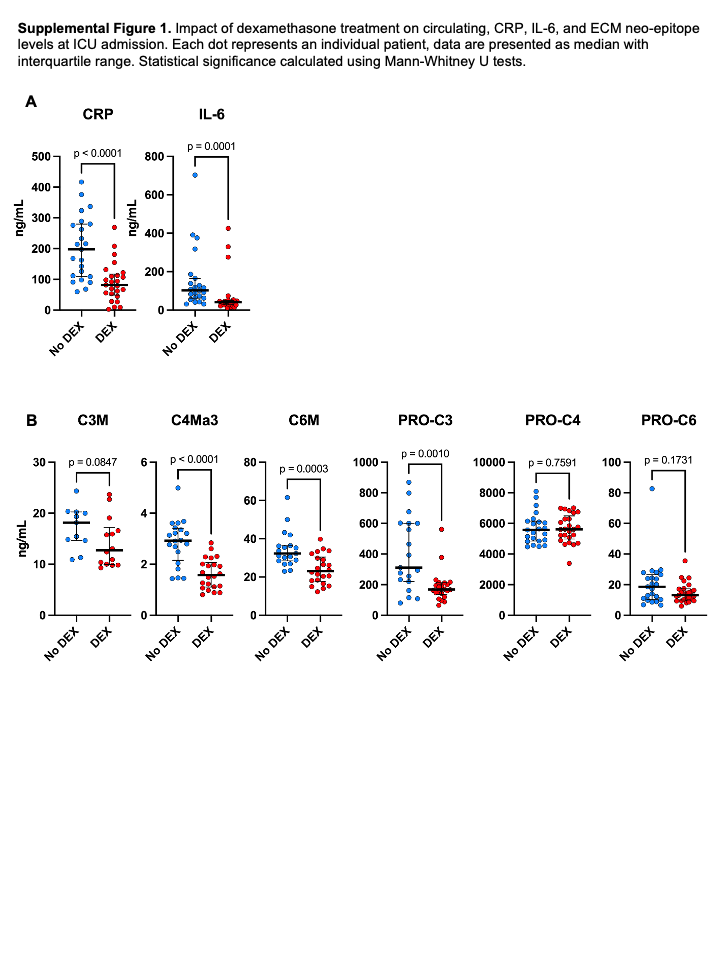

Supplement: Supplementary file 1 — Supplementary Material 1 [file 12931_2025_3098_MOESM1_ESM.png]
